# Effect of Bone Marrow Mesenchymal Stem Cell Sheets on Skin Capillary Parameters in a diabetic wound model: A Novel Preliminary Study

**DOI:** 10.52547/ibj.25.5.334

**Published:** 2021-08-28

**Authors:** Maryam Habibi, Farzaneh Chehelcheraghi

**Affiliations:** 1 Student Research Committee, School of Medicine, Lorestan University of Medical Sciences, Khorramabad, Iran;; 2 Department of Anatomical Sciences, School of Medicine, Lorestan University of Medical Sciences, Khorramabad, Iran

**Keywords:** Neoangiogenesis, Skin flap, Transplantation, Wound healing

## Abstract

**Background:**

Treatment with BMMSCs has anti-inflammatory, tissue regenerative, angiogenic, and immune-stimulating effects. When using as sheets or accumulate, BMMSCs causes the development of neoangiogenesis in damaged skin tissue. Diabetes, a metabolic disorder, can negatively affect many physiological functions, including the process of skin injury repair. This adverse impact may increase the risk of skin surgery. RSF is commonly used in reconstructive surgery. The terminal part of the RSF is often affected by necrosis because of impaired blood flow, which is exacerbated in diabetes. This study investigated the effect of stem cells, applied as accumulated or cell sheets, along with RSF surgery on skin capillaries in STZ-induced diabetic rats.

**Methods:**

Thirty male Wistar rats were divided into three groups (n = 10): diabetes-RSF control, diabetes-RSF local applied stem cells (loc-BMMSCs), diabetes-RSF applied stem cells as accumulated or cell sheets (ac-BMMSCs). Two weeks after the STZ injection, RSF surgery and stem cell therapy (6 × 10^9^) were carried out (day zero). Furthermore, stereological methods were used to investigate the capillary patterns among the groups. Anti-CD31/PCAM1 immunohistochemistry was also used for further confirmation of changes in capillary parameters.

**Results:**

The results demonstrated that capillaries were protected by MSC sheets in the flap tissue, and the thickness of the epidermal layer was improved, indicationg the possible beneficial effects of MSC sheets on diabetic wound treatment.

**Conclusion:**

Stem cells, as ac-BMMSCs, may decrease the levels of wound healing complications in diabetes and can be considered as a cell therapy option in such conditions.

## INTRODUCTION

Stem cell therapy accelerates the wound healing process in chronic wounds. Due to the weakened immune system in DM, most wounds become infected^[^^1^^]^. RSFs are commonly used in plastic surgery of various types of chronic wounds, including diabetic wounds^[^^2^^]^. Despite using skin flaps in the treatment of different skin injuries, flap necrosis is one of the most important complications after reconstructive and plastic surgery. As the blood supply exists only in the flap’s base, the distal part is more susceptible to ischemia and necrosis^[^^3^^]^. In this sense, it is crucial to determine the size and ratio of the length to the width of the flap^[^^4^^]^. Distal flap necrosis results in secondary surgery, which may in turn delay the subsequent treatments. However, plastic surgeons take a number of steps to improve the skin flap survival. Therefore, besides advanced technologies, the effectiveness of therapy is of paramount importance. 

One of the key factors in wound healing is the blood flow enhancement through the improvement of vascular conditions or neoangiogenesis within the tissue, which is especially demanding in diabetic skin ulcers. In recent years, studies have focused mainly on the administration of growth factors and the induction of angiogenesis by gene transfection^[^^5^^]^. Stem cell therapy is a method of accelerating angiogenesis in ischemic tissues^[^^6^^,^^7^^]^. Stem cells possess both the ability to differentiate into multiple cell lineages and the potential for secreting various growth factors and/or cytokines, exerting a beneficial therapeutic effect on the ischemic tissue^[^^8^^-^^10^^]^. 

Surveys have indicated that MSCs may effectively improve the survival of ischemic skin flaps. For instance, the topical delivery of mouse fat-derived MSCs to RSF resulted in a significant increase in flap survival^[^^11^^,^^12^^]^. Stem cells have been indicated to stimulate neoangiogenesis through paracrine mechanisms in ischemic tissues^[^^11^^,^^12^^]^. However, the implantation of MSCs in ischemic tissues is severely restricted due to poor survival^[^^13^^]^. Conventional stem cell admimistration methods, which are sprayed or topically injected, have many challenges and problems owing to the heterogeneity of the cell suspension and the lack of control at the injection site^[^^14^^]^. 

New engineering technologies, such as cell compression engineering, can prevent such hazards by preserving the extracellular matrix, cell-to-cell connections, and cell matrix connections. As a result, the transplanted cells are preserved at the transplant site^[^^15^^]^. *In vivo* studies have already proven that cell sheets can provide a better long-term tissue survival as compared to the injection of a cell suspension^[^^16^^]^. Among all cell types used in angiogenesis studies, endothelial cells have widely been applied in different types of matrices to produce vascular networks^[^^17^^,^^18^^]^. Endothelial cells, when cultured with human MSCs, increase the release of angiogenic growth factors, which enhances the maturation and stability of newly formed vascular structures^[^^19^^]^. Therefore, engineered cell sheets, if used around vessels, are more effective than other sites employed in full-thickness skin lesions^[^^20^^]^. Hence, prevascularized cell sheets or compact seem to improve skin flap survival by accelerating local neoangiogenesis. 

The aim of this study was to evaluate the inductive effect of BMMSCs cell sheets or accumulated cells on healing process of diabetic wounds, neovascularization and increased skin flap survival.

## MATERIALS AND METHODS

**Animals**

The study was conducted on 30 three-month-old male Wistar rats (300 ± 50 g). The rats were housed individually in hygienic cages with food and water available *ad libitum* at the temperature of 22 ± 2°C and the humidity of 55 ± 10% with a constant 12 h-light/12 h-darkness cycle. All surgical procedures were performed under sterile conditions.

**Isolation and identification of BMMSCs**

The isolation and primary culture procedure of BMMSC have previously been reported^[^^21^^]^. In brief, the freshly collected BMMSCs were planted onto 90-mm culture plates containing α-MEM medium complemented by 2.5 mM of glutamine, 90 U/ml of penicillin, and 90 mg/ml of streptomycin. The third passage of BMMSCs was used in flow cytometry analysis and documentation of stem cell features. The procedure of flow cytometry analysis was similar to the one previously reported^[^^22^]. In brief, surface markers CD90, CD45, CD34, and CD105 were characterized by Iranian Biological Resource Center, Tehran, Iran (code number; IB RCC10164). Following the multilinage differentiation of MSC, the osteogenic differentiation was induced in an α-MEM medium containing 0.1 μM of dexamethazone, 50 μM of ascorbic acid, and 10 mM of glycerophosphate. Besides, adipogenic differentiation was induced in α-MEM with 0.5 μM of dexamethazone, 0.5 mM of 3-isobutyl-1-methylxanthine, and 0.1 mM of indomethacine^[^^23^^,^^24^^]^.

**Cell accumulation and single cell suspension preparation**

BMMSCs of the third passage were used to prepare BMMSC accumulation. The cells were seeded at a density of 6 × 10^9^/cm^2^ on 50-mm diameter culture dishes in a humidified atmosphere containing 5% CO_2_ at 37 °C. At a confluence rate of 80-90%, DMEM condition medium was exchanged and the incubation was carried out in the presence of 90 mg/ml of vitamin C and 10 nM of dexamethasone until a white layer structure was observed. The cultivation was continued for 12 days to make the accumulation thicker. BMMSC suspension was then enumerated by an electronic cytometer for the average cell number in one accumulation. Then the same number of the third passage of BMMSCs was prepared into a single cell suspension for *in vivo* administration experiments. BMMSCs were also labeled with fluorescence molecule (DiI cell-labeling solution)^[^^25^^]^.

**Induction of DM**

DM was induced by STZ (dissolved in sterile sodium citrate buffer, 50 mM, pH 4.7). Each rat received a dose of 80 mg/kg by intraperitoneal injection of 600 µL of STZ. DM was verified when fasting blood glucose levels were higher than 250-300 mg/dL. The diabetic animals were maintained for 30 days to ensure the establishment of DM.

**Ischemic RSF model and cell accumulation implantation**

All the rats were randomly divided into three groups (n = 10 in each group). The control group was left untreated. The local cell group (loc-BMMSCs) received a subdermal injection of the BMMSCs (volume; 6 × 10^9^ in 1.0 ml DMEM) at eight points on each flap, administered once immediately after surgery. The accumulated cell group (ac-BMMSCs) received the cells in the same place and date as in loc-BMMSCs but in form of engineered sheets. All the animals were anesthetized intramuscularly with ketamine hydrochloride (50 mg/kg) and diazepam (5 mg/kg)^[^^2^^]^. After the rats’ skin was shaved, RSFs, including the entire thickness of the skin and skin muscle (panniculus carnosus), were made. The base of the RSFs was located at the distal end of the animal on a horizontal line between the crest of the iliac bones. The dimension of the flaps was 1.5 × 7.5 cm (width:length 1:5). After lifting the tissue and cutting all the vascular connecting, it was returned to its original location and sutured using interrupted 4/0 nylon sutures. The day of surgery was considered as day zero. Immediately seven days after surgery, the surface area of the flap was photo captured and measured by Image J^[^^26^^]^.

**Assessment of skin flap surviving areas and clinical examination**

**Fig. 1:**
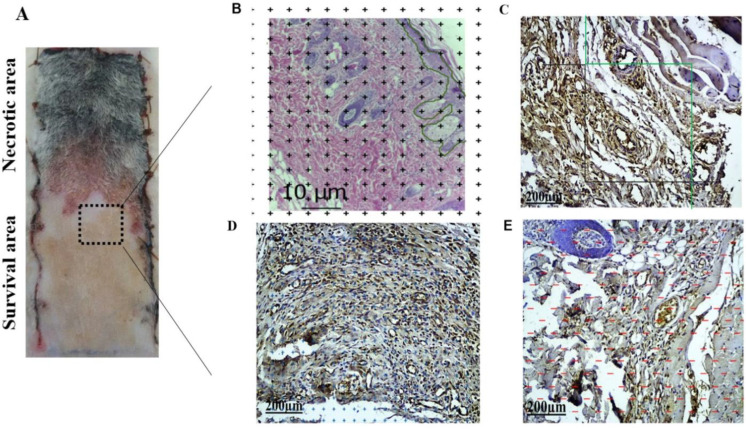
Graphical explanation of stereology techniques. (A) Flap tissue at day seven. H&E staining was used to reveal the skin partition; (B) epidermal layer marked by a green line (scale bar = 10 µm); C, D, and E represent immunostained tissues using anti-PECAM-1 antibody. (C) the computation technique of capillary length. The calculation method of capillary length was as follows: each captured image was put on an unbiassed counting frame. The only vessel in the frame and to touch the black line were considered; (D) The calculation method of capillary volume, the number of collisions per capillary with the in-frame algorithm was calculated. (E) The calculation method of capillary surface area, the area employed by the capillaries was also estimated in a particular frame by placing them on the pattern lines (scale bar = 200 µm)

All rats were sacrificed by inhaling chloroform on day seven after surgery. Color, texture, edema, hair growth, and necrosis area were observed and evaluated in three groups. If the flap became black and withered, with no bleeding after a needle inserion, it was diagnosed as necrosis, and the rest of the flap area was considered as surviving area. Samples were taken from the survival areas to evaluate histological parameters (Fig. 1A)^[^^26^^]^. After then, the skin tissues were fixed using 4% paraformaldehyde, and 5 μm-thick sections were prepared and stained with H&E method and examined by stereological methods.

**Assessment of epidermal volumes in flap survival region**

H&E staining was performed to mark the epidermal surface area in flap survival region (Fig. 1A^[^^27^^]^). The calculation was as follows ^[^^28^^]^: V (epi) = t × a (p) × ∑p (epi) × 2. Where *t* signifies the slab thickness (1 mm), *a* stands for the area associated with each point, and ΣP (epi) refers to the total number of points hitting the V (epi) in flap survival region. Calibration was performed using a checkerboard pattern on a paper (Fig. 1B).

**Immunohistochemistry**

The prepared slabs were embedded in paraffin, and the sector method was employed to generate isotropic and uniformly random slices^[^^29^^]^. From each animal, 20 samples with a width of 5 µm were collected. 

**Immunostaining** **technique**

The following steps were performed on each slide, respectively. First, the slide was fixed in acetone for 10 minutes and then placed in citrate solution at high temperature for 15 min. To inactivate the intra-tissue peroxidase, the slide was exposed to 3% hydrogen peroxide at 37 °C for 15 minutes, and nonspecific staining was blocked by normal goat serum at 37 °C for 30 min. The mouse monoclonal anti-CD31 antibody, also known as PECAM-1, was selected and used for immunolabelling at 4 °C overnight. Dyinobenzidine solution was used to visualize the color. The largest size of of the selected capillaries was 10 μm in diameter^[^^30^^]^ (Fig. 1C, 1D, and 1E).

**Total length of capillaries in flap survival region**

All images were evaluated randomly for capillary structures (Fig. 1C). The following formula was applied^[^^31^^]^: 

LV (^cap^/_SR_) = 2×∑Q (^cap^/_SR_)/∑A

ΣQ (^cap^/_SR_) means the total number of capillary profiles in survival region counted per rat. ΣA indicates the total area of the counting frames used per rat. The total length of the capillaries in the survival region could be obtained by multiplying the length density of the capillaries in SR, LV (^cap^/_SR_), by the SR volume (V (SR)). 

**Total volume of capillaries in flap survival region**

Every captured picture was randomly placed by a transparent point grid (Fig. 1D). The volume fraction of the capillaries in the SR, VV (cap/SR), was analyzed using the following formula^ [^^31^^]^: VV (^cap^_/SR_) = ΣP (^cap^_/SR_)/ ΣP. The total volume of the capillaries in the SR was analyzed by multiplying the volume fraction of the capillaries in the SR, VV (^cap^/_SR_), by the SR volume (V(SR)).

**Total area of capillaries in SR**

The area employed by capillaries in SR was estimated by the following equation (Fig. 1D)^[^^31^^]^: SV (^cap^/_SR_) = 2 × ΣL (^cap[^^32^^]^)/ΣL*. *The SV (cap (SR)) expansion through the volume of SR (V (SR)) was used. 

**Data analysis**

GraphPad Prism 8 software was used to statistically analyze the data. One-way analysis of variance (ANOVA) or multivariate analysis of variance was performed by post hoc Tukey method for direct comparison. Data were reported as the mean ± standard deviation of the mean. *p* < 0.05 was considered as statistically significant.

**Ethical statement**

The above-mentioned sampling protocols were approved by the Medical Ethics Committee of Lorestan University, Lorestan, Iran (ethical code: IR.LUMS.REC.1397.111). 

## RESULTS

**Blood glucose** **concentration evaluation**

**Fig. 2:**
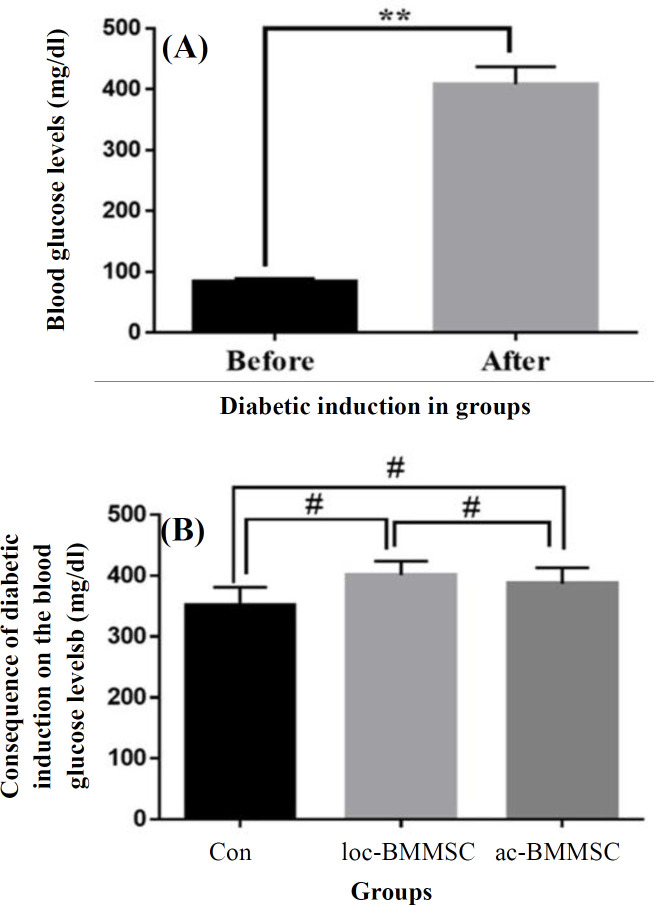
(A) Changes of blood glucose levels before and after diabetic induction. (B) The comparison of blood glucose levels between before and after diabetic induction in the cell recipents and control group. The results of diabetic induction in blood glucose levels are shown in three groups (^*^p < 0.05 and ^#^*p* > 0.05)

Blood glucose concentration was 84 ± 5 (in a range of 66-98) mg/dl before diabetes induction, but following the induction, the glucose levels reached 408 ± 29 (in a range of 300-600) mg/dl in the control groups and cells recipient groups, which were significantly higher than non-diabetic status (*p* = 0.02; Fig. 2A). Also, abnormal high glucose levels were detected in the three groups, and there was no statistically significant difference between the three groups) *p* = 0.32; Fig. 2B).

**DiI + BMMSC accumulation and its specifications**

**Fig. 3:**
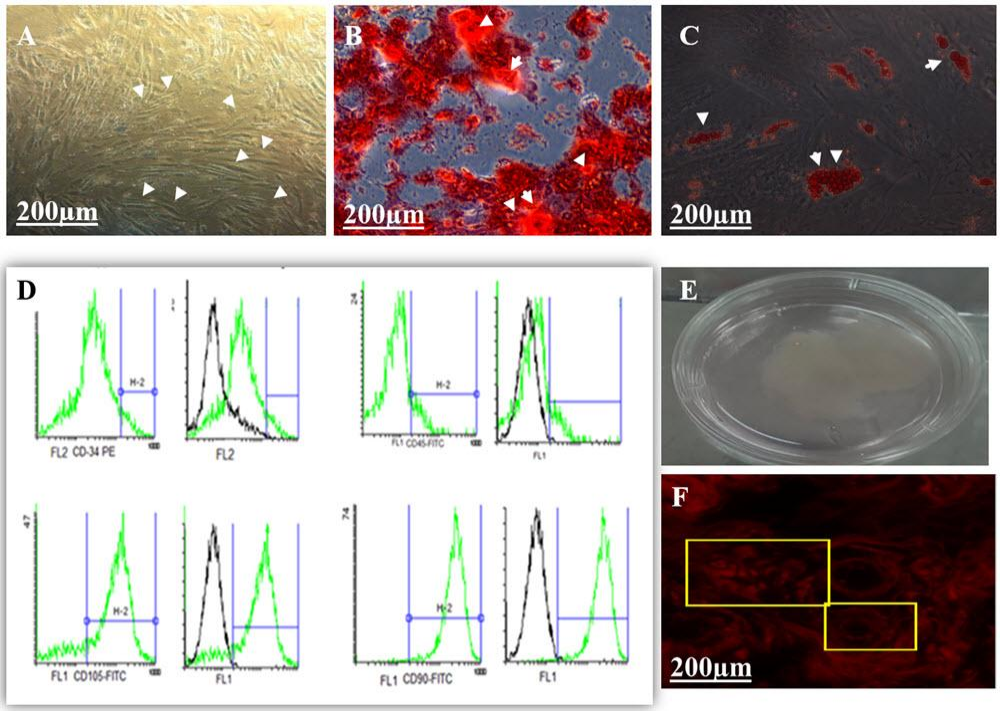
DiI + BMMSC accumulate construction and expression characteristic. (A, B, and C) Multi-differentiation of BMMSCs. The third passage of BMMSCs was used to determine multi-differentiationfor control (A), osteogenesis (B), or adipogenesis (C), and the characterization of a cluster of differentiations (CDs) of BMMSCs. (D Positive expression of mesenchymal stem cell markers CD105 and CD90 and negative expression of the hematopoietic markers CD45, CD34. The whole aggregate was scratched off the dish (E). In accumulation and under the excitation of 408 nm fluorescence, DiI + BMMSCs produced red light (F)

Cells were cultured under standard conditions. The process of bone and fat differentiation was induced, and the specific phenotype changes were observed, exhibiting fibroblast-like morphology (Fig. 3A). The presence of CD105 and CD90 MSCs markers and the absence of CD45 and CD34 hematopoietic markers were confirmed by flow cytometry (Fig. 3D). BMMSCs were incubated with vitamin C and dexamethasone for 12 days. Then the densely packed cells (sheet or memmbrane) were visible in the bottom of the container. A cluster description of the BMMSC differentiation (CD) was observed, as well (Fig. 3C). Electronic cytometer revealed an average number of 1.24 ± 0.06 × 10^6^ cells for one accumulation. Accordingly, single cell suspension containing 1.24 × 10^6^ DiI + BMMSC was prepared in saline solution for administration. Dil-labeled cells emitted red fluorescence on the seventh day after surgery (408 nm; Fig. 3F), which could be seen with a fluorescence microscope.

**Assessment of skin flap surviving areas and clinical examination**

The blood supply of the RSF mainly depends on the distance from the base of the flap. At seven days, after the tissue in skin flaps was elevated, the skin flaps showed symptoms of visible purple color. We observed the color change and hair growth on the skin flaps at the time point of day seven (Fig. 1A). 

**Increased volumes of the epidermal of the flap survival region by ac-BMMSC**

**Fig. 4:**
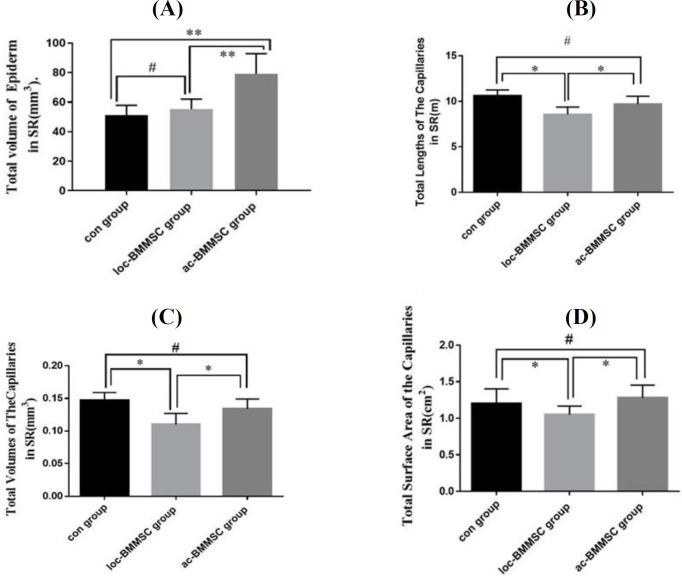
Inﬂuence of BMMSCs on the histology parameters of the flap survival regions. (A) Evaluation of the total volume of epiderm; (B) evaluation of the capillaries’ total lengths; (C) comparison of the capillaries’ total volume; (D) comparison of the capillaries’ total surface area in the flap survival region among the control group, loc-BMMSC group and ac-BMMSC group (^∗^Reveals *p* < 0.05, ^#^ p>0.05)

The total volume of epidermis in flap survival regions were 51 ± 6.9 mm^3^ in the control group, 79.2 ± 13.66 mm^3^ in the ac-BMMSC group, and 55.33 ± 6.74 mm^3^ in the loc-BMMSC group at seven days post surgery. Total epidermal volume increased in Ac-BMMSC group compared to control group, but it was not significant (*p* = 0.121). Also, the total volume of epidermis in ac-BMMSC group was significantly increased (*p* = 0.01) compared to that of Loc-BMMSC (Fig. 4A).

**Variations in the total parameters of the capillaries in flap survival region**

Stereological methods were used to evaluate capillary parameters in different BMMSCs treated groups. To this end, immunostaining of skin tissues using anti-PECAM-1 antibody was also carried out to further confirm the morphological evidence (Fig. 1C, 1D, and 1E). The total lengths of the capillaries in the flap survival region were 10.6 ± 0.64 cm^2^ in the control group, 8.56 ±0.81 cm^2^ in the loc-BMMSC group, and 9.7 ±0.865 cm^2^ in the ac-BMMSC group. Accumulation of BMMSC significantly improved the total length of the capillaries in the flap survival region compared to the loc-BMMSC group (Fig. 4B). The total volumes of the capillaries in the survival region were 0.147 ± 0.0120 cm^2^ in the control group, 0.110 ± 0.0170 cm^2^ in the loc-BMMSC group, and 0.134 ± 0.0150 cm^2^ in the ac-BMMSC group. Acumulation of BMMSC significantly increased the total volumes of the capillaries in the flap survival region in ac-BMMSC group compared with that of the loc-BMMSC group (Fig. 4C). The total surface areas of the capillaries in the survival region were 1.2 0 ± 0.203cm^2^ in the control group, 1.05 ± 0.116 cm^2^ in the loc-BMMSC group, and 1.28 ± 0.174 cm^2^ in the ac-BMMSC group. Accumulation of BMMSC meaningfully improved the total surface area of the capillaries in the survival region in the ac-BMMSC group compared with that in the loc-BMMSC group (Fig. 4D). There was no significant difference between the control group and the ac-BMMSC group in all three parameters assessed in the flap survival region (*p* > 0.05).

## DISSCUSION

With the development of stem cell technology, therapeutic approaches in diabetic wound healing have advanced^[^^9^^]^. One of the new methods of using stem cells in reconstructive medicine is the accumulated form of these cells, which has been used with great success in the treatment of limb wounds^[^^33^^]^. The findings from this study indicated that the administration of cell accumulation could improve skin flap survival in ac-BMMSCs group. This effect may be related to the promotion of angiogenesis and the reduction of inflammation response. The extraction and exudation of growth factors have been reported to be an essential function of BMMSCs, and different regenerative effects of BMMSCs in the skin have already been demonstrated^[^^34^^]^. On the other hand, the quality of animal models is the key to the success of *in vivo* experiments. We decided to use the RSF to avoid, as far as possible, experimental errors, which is likely due to the supplementary blood supply from adjacent tissues and capillaries in skin flaps.

We demonstrated that ac-BMMSCs could modulate inflammation and granulation tissue, as well as stimulating better vascular growth at seven days post operation in diabetic conditions. There was also no evidence of differentiation of BMMSCs as accumulated cells *in vivo*, which is revealed by DiI fluorescent staining. However, BMMSCs may accelerate wound healing by secreting a large amount of vascular endothelial growth factor A. Studies have demonstrated that the adminiatration of BMMSCs in wound area may increase the levels of specific factors on day seven, helping wound healing^[^^35^^]^. Vascular endothelial growth factor is an necessary elements for migration^[^^36^^]^ and proliferation of endothelial cells^[^^37^^]^. As the number of cytokines increases, more inflammation occurs in the wound area. Following the inflammation, BMMSCs exert their immune-regulating properties and affect the wound site^[^^38^^]^. An earlier research has shown the extent of cell loss during or after transplantation at the wound site. The aggregated forms,can provide a sufficient amount of the cells in the wound healing microenvironment^[^^39^^]^. In the present study, the volume of epidermal layer of flap survival region, total length, total dimensions, and total surface area of the vessels in flap survival region were compared among different groups^[^^40^^]^. Researchers have suggested that overcoming vascular disorders is one of the most essential treatment options for diabetic wounds^[^^41^^]^ and diabetic ulcers and vascular disorders have similar challenges^[^^42^^]^. We assumed that inducing more blood flow at the site of these types of wounds may improve the repairing process. After BMMSC administration, the vascular density augmented as a result of the metabolic need^[^^43^^]^. Vessel distribution in the skin is enormously unequal, and the whole position of capillaries cannot be represented by two-dimensional information on the surface such as thickness. New stereological methods are introduced to gain true three-dimensional construction morphometric structures^[^^28^^]^. 

In summary, administering stem cells in form of ac-BMMSCs and allowing cells to migrate separately to the impaired area are beneficial in cell therapies. Cell sheet of BMMSCs using a scaffold could be a safer and more effective therapeutic modality for various systemic diseases. Stem cell sheets also enhance the possibility of inexpensive treatments. However, the description of active factors involved in wound healing and BMMSC paracrine functions have been in the early stages due to the low sensitivity of the analysis of these factors. Therefore, identifying the active agents of ac-BMMSC would be the next goal of our research. It seems that these possible potential factors will deeply affect the future strategies in regeneration medicine.

## References

[1] Berlanga-Acosta J, López-Mola E, Garcia-Siverio M, Guillén-Nieto G, López-Saura P, Valdez-Pérez C, Puentes-Madera I, Savigne-Gutierrez W, Álvarez-Duarte H, Miranda-Espinosa N (2012). Detrimental impact of acute and chronic glucose burden in wound-healing cells: fibroblasts, myofibroblasts and vascular precursor cells. Biotecnología aplicada journal.

[2] Zhao L, Lu J, Wang C, Zhao W, Qing E, Ma J (2013). Prostaglandin E1 increases the blood flow rate of saphenous vein grafts in patients undergoing off-pump coronary artery bypass grafting. Journal of cardiothoracic and vascular anesthesia.

[3] Kiumehr S, Demehri S, Rabbani S, Amanpour S, Mohagheghi MA, Dehpour AR (2005). Preconditioning of the rat random-pattern skin flap: modulation by opioids. British journal of plastic surgery.

[4] Miyawaki T, Jackson IT, Bier UC, Andrus L, Williams F, Bradford M (2001). The effect of capsaicin ointment on skin for the survival of a cutaneous flap. European journal of plastic surgery.

[5] Gurunluoglu R, Ozer K, Skugor B, Lubiatowski P, Carnevale K, Siemionow M (2002). Effect of transfection time on the survival of epigastric skin flaps pretreated with adenovirus encoding the VEGF gene. Annals of plastic surgery.

[6] Raval Z, Losordo DW (2013). Cell therapy of peripheral arterial disease: from experimental findings to clinical trials. Circulation research.

[7] Boyce ST, Lalley AL (2018). Tissue engineering of skin and regenerative medicine for wound care. Burns and trauma.

[8] Kinnaird T, Stabile E, Burnett MS, Shou M, Lee CW, Barr S, Fuchs S, Epstein SE (2004). Local delivery of marrow-derived stromal cells augments collateral perfusion through paracrine mechanisms. Circulation.

[9] Wang T, Tang W, Sun S, Wan Z, Xu T, Huang Z, Weil MH (2009). Mesenchymal stem cells improve outcomes of cardiopulmonary resuscitation in myocardial infarcted rats. Journal of molecular and cellular cardiology.

[10] Sasaki M, Abe R, Fujita Y, Ando S, Inokuma D, Shimizu H (2008). Mesenchymal stem cells are recruited into wounded skin and contribute to wound repair by transdifferentiation into multiple skin cell type. The journal of immunology.

[11] Lee DW, Jeon YR, Cho EJ, Kang JH, Lew DH (2014). Optimal administration routes for adipose‐derived stem cells therapy in ischaemic flaps. Journal of tissue engineering and regenerative medicine.

[12] Uysal AC, Mizuno H, Tobita M, Ogawa R, Hyakusoku H (2009). The effect of adipose-derived stem cells on ischemia-reperfusion injury: immunohistochemical and ultrastructural evaluation. Plastic and reconstructive surgery.

[13] Yanagawa B, Kataoka M, Ohnishi S, Kodama M, Tanaka K, Miyahara Y, Ishibashi-Ueda H, Aizawa Y, Kangawa K, Nagaya N (2007). Infusion of adrenomedullin improves acute myocarditis via attenuation of myocardial inflammation and edema. Cardiovascular research.

[14] Lin YD, Yeh ML, Yang YJ, Tsai DC, Chu TY, Shih YY, Chang MY, Liu YW, Tang ACL, Chen TY (2010). Intramyocardial peptide nanofiber injection improves postinfarction ventricular remodeling and efficacy of bone marrow cell therapy in pigs. Circulation.

[15] Li M, Ma J, Gao Y, Yang L (2019). Cell sheet technology: a promising strategy in regenerative medicine. Cytotherapy.

[16] Moschouris K, Firoozi N, Kang Y (2016). The application of cell sheet engineering in the vascularization of tissue regeneration. Regenerative medicine.

[17] Heinrich AC, Patel SA, Reddy BY, Milton R, Rameshwar P (2009). Multi-and inter-disciplinary science in personalized delivery of stem cells for tissue repair. Current stem cell research and therapy.

[18] Korff T, Augustin HG (1998). Integration of endothelial cells in multicellular spheroids prevents apoptosis and induces differentiation. journal of cell biology.

[19] Jain RK, Au P, Tam J, Duda DG, Fukumura D (2005). Engineering vascularized tissue. Nature biotechnology.

[20] Chen L, Xing Q, Zhai Q, Tahtinen M, Zhou F, Chen L, Xu Y, Qi S, Zhao F (2017). Pre-vascularization enhances therapeutic effects of human mesenchymal stem cell sheets in full thickness skin wound repair. Theranostics.

[21] He X, Dong Z, Cao Y, Wang H, Liu S, Liao L, Jin Y, Yuan L, Li B (2019). MSC-derived exosome promotes M2 polarization and enhances cutaneous wound healing. Stem cells international.

[22] Rezazadeh K, Chehelcheraghi F, Anbari K (2018). The effect of bone marrow derived mesenchymal stem cells on the survival of random skin flap on sterptozotocin-induced diabetic rats. Journal of advances in medical and biomedical research.

[23] Sekine H, Shimizu T, Matsuura K, Yamato M, Takahashi M, Murakami T, Kobayashi EK, Hagiwara N, Okano T (2009). Cell sheet transplantation improves damaged heart function via more cell survival in comparison with dissociated cell injection. Circulation.

[24] Wu Y, Chen L, Scott PG, Tredget EE (2007). Mesenchymal stem cells enhance wound healing through differentiation and angiogenesis. Stem cells.

[25] Sui BD, Hu CH, Liu AQ, Zheng CX, Xuan K, Jin Y (2019). Stem cell-based bone regeneration in diseased microenvironments: challenges and solutions. Biomaterials.

[26] Chai J, Ge J, Zou J (2019). Effect of autologous platelet-rich plasma gel on skin flap survival. Medical science monitor.

[27] Alex JC, Bhattacharyya TK, Smyrniotis G, O'Grady K, Konior RJ, Toriumi DM (2001). A histologic analysis of three‐dimensional versus two‐dimensional tissue expansion in the porcine model. Laryngoscope.

[28] Howard CV, Cruz‐Orive LM, Yaegashi H (1992). Estimating neuron dendritic length in 3D from total vertical projections and from vertical slices. Acta neurologica scandinavica.

[29] Wobser M, Siedel C, Schrama D, Bröcker EB, Becker JC, Vetter-Kauczok CS (2006). Expression pattern of the lymphatic and vascular markers VEGFR-3 and CD31 does not predict regional lymph node metastasis in cutaneous melanom. Archives of dermatological research.

[30] Mendis KR, Balaratnasingam C, Yu P, Barry CJ, McAllister IL, Cringle SJ, Yu DY (2010). Correlation of histologic and clinical images to determine the diagnostic value of fluorescein angiography for studying retinal capillary detail. Investigative ophthalmology and visual science.

[31] Mathieu C, Chevrier A, Lascau-Coman V, Rivard GE, Hoemann CD (2013). Stereological analysis of subchondral angiogenesis induced by chitosan and coagulation factors in microdrilled articular cartilage defects. Osteoarthritis and cartilage.

[32] Sridharan G, Shankar AA (2012). Toluidine blue: a review of its chemistry and clinical utility. Journal of oral and maxillofacial pathology.

[33] Barrientos S, Stojadinovic O, Golinko Ms, Brem H, Tomic‐Canic M (2008). Growth factors and cytokines in wound healing. Wound repair and regeneration.

[34] Yeum CE, Park EY, Lee SB, Chun HJ, Chae GT (2013). Quantification of MSCs involved in wound healing: use of SIS to transfer MSCs to wound site and quantification of MSCs involved in skin wound healing. Journal of tissue engineering and regenerative medicine.

[35] Gnecchi M, He H, Liang OD, Melo LG, Morello F, Mu H, Noiseux N, Zhang L, Pratt RE, Ingwall JS (2005). Paracrine action accounts for marked protection of ischemic heart by Akt-modified mesenchymal stem cells. Nature medicine.

[36] Xue W, Mizukami I, Todd RF, Petty HR (1997). Urokinase-type plasminogen activator receptors associate with β1 and β3 integrins of fibrosarcoma cells: dependence on extracellular matrix components. Cancer research.

[37] Gerber H-P, Dixit V, Ferrara N (1998). Vascular endothelial growth factor induces expression of the antiapoptotic proteins Bcl-2 and A1 in vascular endothelial cells. Journal of biological chemistry.

[38] Jiao J, Milwid JM, Yarmush ML, Parekkadan B (2011). A mesenchymal stem cell potency assay. Methods in molcular biology.

[39] Badiavas EV, Abedi M, Butmarc J, Falanga V, Quesenberry P (2003). Participation of bone marrow derived cells in cutaneous wound healing. Journal of cellular physiology.

[40] Najar M, Rouas R, Raicevic G, Boufker HI, Lewalle P, Meuleman N, Bron D, Toungouz M, Martiat P, Lagneaux L (2009). Mesenchymal stromal cells promote or suppress the proliferation of T lymphocytes from cord blood and peripheral blood: the importance of low cell ratio and role of interleukin-6. Cytotherapy.

[41] Nakatani D, Sato H, Sakata Y, Shiotani I, Kinjo K, Mizuno H, Shimizu M, Ito H, Koretsune Y, Hirayama A (2005). Influence of serotonin transporter gene polymorphism on depressive symptoms and new cardiac events after acute myocardial infarction. American heart journal.

[42] Rennert RC, Sorkin M, Januszyk M, Duscher D, Kosaraju R, Chung MT, Lennon J, Radiya-Dixit A, Raghvendra S, Maan ZN, Hu MS, Rajadas J, Rodrigues M, Gurtner GC (2014). Diabetes impairs the angiogenic potential of adipose-derived stem cells by selectively depleting cellular subpopulations. Stem cell research and therapy.

[43] Kuo YR, Wang CT, Cheng JT, Wang FS, Chiang YC, Wang CJ (2011). Bone marrow–derived mesenchymal stem cells enhanced diabetic wound healing through recruitment of tissue regeneration in a rat model of streptozotocin-induced diabetes. Plastic and reconstructive surgery.

